# Factors Involved in the Apoptotic Cell Death Mechanism in Yellow Fever Hepatitis

**DOI:** 10.3390/v14061204

**Published:** 2022-06-01

**Authors:** Jeferson da Costa Lopes, Luiz Fábio Magno Falcão, Arnaldo Jorge Martins Filho, Marcos Luiz Gaia Carvalho, Caio Cesar Henriques Mendes, Fábio Alves Olímpio, Vanessa do Socorro Cabral Miranda, Lais Carneiro dos Santos, Jannifer Oliveira Chiang, Ana Cecilia Ribeiro Cruz, Vanessa Costa Alves Galúcio, Raimunda do Socorro da Silva Azevedo, Lívia Caricio Martins, Maria Irma Seixas Duarte, Jorge Rodrigues de Sousa, Pedro Fernando da Costa Vasconcelos, Juarez Antônio Simões Quaresma

**Affiliations:** 1 Evandro Chagas Institut, Ministry of Health, Ananindeua 67015-120, Brazil; jefersonchaz@hotmail.com (J.d.C.L.); arnaldofilho@iec.gov.br (A.J.M.F.); marcosgaia@outlook.com (M.L.G.C.); vanessacabralmiranda@gmail.com (V.d.S.C.M.); laiscarneiros@gmail.com (L.C.d.S.); janniferchiang@iec.gov.br (J.O.C.); anacecilia@iec.gov.br (A.C.R.C.); raimundaazevedo@iec.gov.br (R.d.S.d.S.A.); liviamartins@iec.gov.br (L.C.M.); krekrodrigues@gmail.com (J.R.d.S.); pedrovasconcelos@iec.gov.br (P.F.d.C.V.); 2 Department of Pathology, State University of Para, Belem 66050-540, Brazil; fabiofalcao@uepa.br (L.F.M.F.); caio_henriques12@hotmail.com (C.C.H.M.); vanessagalucio@gmail.com (V.C.A.G.); 3 Tripical Medicine Unit, Federal University of Para, Belem 66075-110, Brazil; f.olimpiomilitar@gmail.com; 4 Schhol of Medicine, Sao Paulo University, Sao Paulo 05508-070, Brazil; miduarte@usp.br

**Keywords:** cell death, flavivirus, pathogenesis, apoptosis, arbovirus

## Abstract

Yellow fever (YF), a non-contagious infectious disease, is endemic or enzootic to the tropical regions of the Americas and Africa. Periodic outbreaks or epidemics have a significant impact on public health. Programmed cell death, or apoptosis, is generally characterised by distinct morphological changes and energy-dependent biochemical pathways. In this study, we performed immunohistochemistry analysis to identify and quantify proteases and protein targets involved in the cascade that triggers apoptosis in YF virus (YFV)-infected human hepatocytes. Liver tissue samples were collected from 26 individuals, among whom 21 were diagnosed as YF-positive, and five were flavivirus-negative and died due to other causes. The histopathological alterations in YFV-positive cases were characterised by the presence of apoptotic bodies, steatosis, cellular swelling, and extensive necrosis and haemorrhage in the hepatic lobules. Additionally, we observed an abundance of inflammatory infiltrates in the portal tract. The expression of various apoptotic markers in the hepatic parenchyma, including CASPASE 3, CASPASE 8, BAX, FAS, FASL, GRANZYME B, and SURVIVIN, differed between YFV-positive cases and controls. Collectively, this study confirmed the complexity of YFV infection-induced apoptosis in situ. However, our data suggest that apoptosis in liver parenchyma lesions may significantly contribute to the pathogenesis of fatal YF in humans.

## 1. Introduction

Yellow fever (YF) is an endemic or enzootic non-contagious infectious disease that is prevalent in the tropical regions of the Americas and Africa. Periodic outbreaks or epidemics have a significant impact on public health. The disease is caused by the YF virus (YFV) (Flaviviridae–Flavivirus), which is vectorised by *Aedes* mosquitoes in Africa and is maintained in wild cycles by non-human primates (NHP) and hematophagous arthropods of the *Haemagogus* and *Sabethes* genera in South America. The re-emergence of the urban cycle in the Americas, which occurs between *Aedes aegypti* and susceptible humans, has been a concern for public authorities owing to the low vaccination coverage in non-endemic and endemic areas [1,2,3,4]. Epidemiologically, fatality in severe cases of YF is estimated to be approximately 31–45%, indicating the public health importance of this disease [5].

After entry into the host, viral replication occurs in the lymph nodes located near the area of the blood meal. The virus is then released into the bloodstream and is transported to multiple organs such as the kidney, heart, spleen, lung, and liver. The liver is the most affected organ in humans [5,6,7].

Among the possible mechanisms involved in the pathogenesis of YFV infection, apoptosis is seemingly crucial in inducing the death of hepatocytes. Apoptosis is a natural physiological process that maintains cell balance by killing undesirable cells. The apoptotic cascade is triggered through extrinsic or intrinsic mechanisms once the host detects a foreign aggressor [8,9]. The host immune response plays a fundamental role in triggering apoptosis, where pro- or anti-inflammatory cytokines contribute to the activation of genes encoding enzymes that participate in apoptosis. The expression of TNF-α and TGF-β could lead to cell cycle dysregulation and activate pro-apoptotic genes that cause apoptosis in parenchymal liver cells. Furthermore, cytokines are abundantly expressed in the areas of the liver with a substantial presence of apoptotic bodies, mainly in the medizonal zone [10,11]. During hepatic impairment, the relationship between the extrinsic and intrinsic pathways of apoptosis is seemingly vital in facilitating immune evasion by pathogens. However, several gaps exist in our understanding of this process, mainly concerning the expression of proteases, the action of pro- and anti-apoptotic regulators *in situ*, and the mechanism by which cell death triggers the destruction of hepatocytes through the interaction of the virus with the host defence system [12]. Thus, we investigated the activation of apoptosis via the extrinsic and intrinsic pathways in situ and its relationship with the development of injury in parenchymal liver cells in fatal human YF cases.

## 2. Materials and Method

### 2.1. Ethics Statement

An analytical cross-sectional study was conducted using retrospective samples from the Pathology Sector of Instituto Evandro Chagas, Ananindeua, Pará, Brazil, from 2001 to 2016. We analysed human liver fragments obtained by viscerotomy, collected from fatal cases, and confirmed the diagnosis by histopathological analysis, immunohistochemistry, and viral molecular biology. Among the 26 cases evaluated, 21 were fatal YFV-positive cases, and five were included in the control group. The cases in the control group showed preservation of the liver parenchyma and tested negative for hepatotropic viruses, based on viscerotomy-derived immunohistochemistry and molecular biology analyses. This study was approved (CAAE 04920918.4.0000.0019) by the Research Ethics Committee (CEP) of the Instituto Evandro Chagas.

### 2.2. Histopathology and Immunohistochemistry

For histopathological analysis, 5 μm sections were obtained from liver fragments embedded in paraffin and stained with haematoxylin–eosin (HE). Tissue immunostaining with specific antibodies against apoptotic markers (Table 1) was performed based on the biotin–streptavidin–peroxidase technique.

Sections were deparaffinised in xylene and hydrated in decreasing ethyl alcohol series (90%, 80%, and 70%). Endogenous peroxidase was blocked with 3% hydrogen peroxide (H_2_O_2_) for 45 min. The antigen was retrieved using citrate buffer (pH 6.0) for 20 min at 90 °C. Nonspecific proteins were blocked with 10% concentrated skim milk for 30 min. Histological sections were then incubated overnight with primary antibodies diluted in 1% bovine serum albumin (BSA).

The slides were then immersed in a 1× phosphate-buffered saline (PBS) and incubated with a biotinylated secondary antibody (labelled streptavidin–biotin (LSAB); DakoCytomation, Glostrup, Denmark) in an oven for 30 min at 37 °C. Subsequently, the slides were immersed in 1× PBS and incubated with streptavidin peroxidase (LSAB; DakoCytomation) for 30 min at 37 °C. The slides were developed using a chromogen solution composed of 0.03% diaminobenzidine (DAB) and 3% H_2_O_2_. The slides were then washed in distilled water and counterstained with Harris haematoxylin for 1 min before being dehydrated in increasing ethyl alcohol series (70%, 80%, and 90%). The slides were then cleaned with xylene.

### 2.3. Quantitative Analysis and Photodocumentation

**Figure 1:**
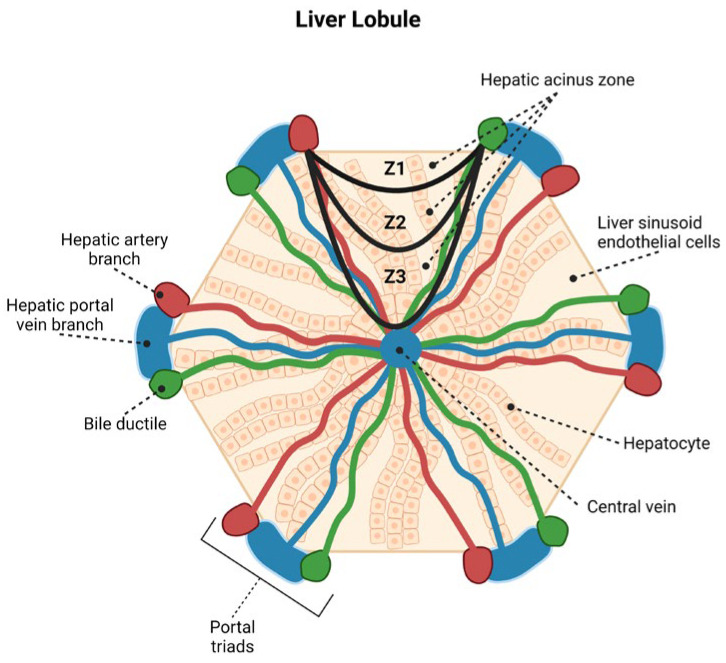
Illustrative diagram demonstrating the hepatic lobule constituted by acini and the division scheme between zone 1 (Z1), zone 2 (Z2), and zone 3 (Z3) located around the portal tract (PT) (Z1), midzonal area (Z2), and around the central lobular vein (Z3). (This figure was created with biorender.com)

Histological analysis was performed using an Axio Imager Z1 microscope (model 456006, Zeiss, Oberkochen, Germany) at 400× magnification. The immunomarkers were quantitatively analysed by selecting 10 fields under 400× magnification in areas I, II, and III of Rappaport and the portal tract (PT) (Figure 1) [8]. Five liver acinar units were detected in each sample, totalling 400 fields in each case. Each field was subdivided into 10 × 10 areas delimited by the microscope lens, comprising an area of 0.0625 mm^2^.

### 2.4. Statistical Analysis

The results obtained were stored in electronic spreadsheets and analysed using the GraphPad Prism 5.0 software. Numerical variables were analysed based on the measures of central tendency and dispersion. The hypotheses were evaluated using ANOVA and Tukey’s post-hoc test. Significance was set at *p* ≤ 0.05.

## 3. Results

### 3.1. Histopathology

**Figure 2:**
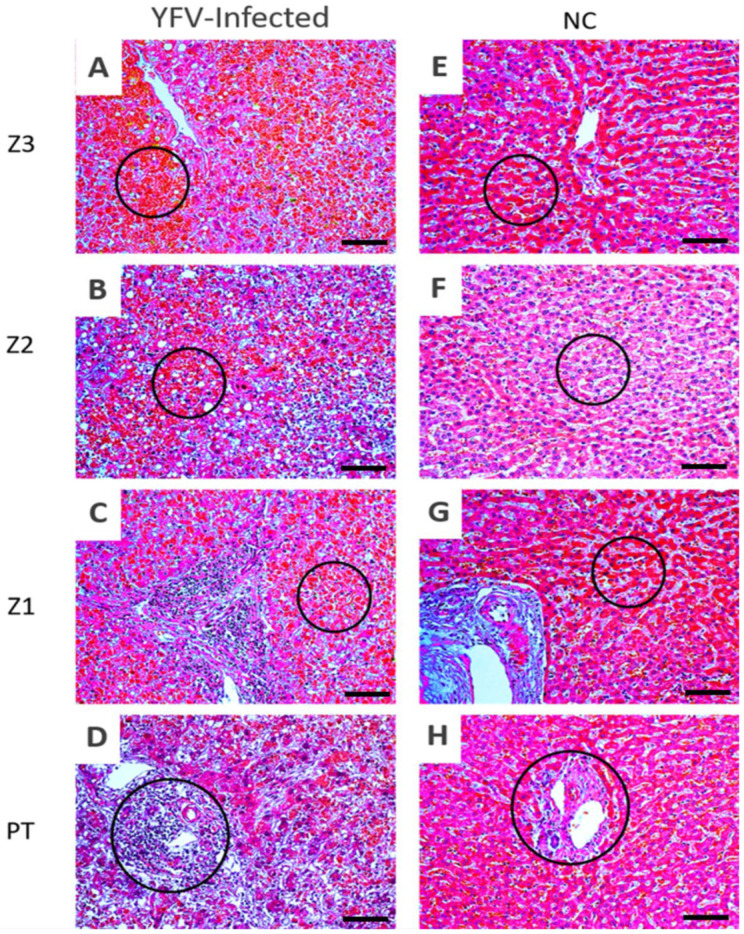
Histopathological analysis of the acinar zones in the liver of humans with fatal yellow fever and normal controls (NC). Z3, Z2, Z1, and PT in the hepatic parenchyma of fatal cases affected by YFV and NC. (**A**–**D**) Necrotic areas with haemorrhagic foci in A-Z3, B-Z2, and C-Z1 (black circles) characterised by areas of presence of red blood cells and absence of nuclei with the presence of cell debris. The massive presence of inflammatory infiltrates in the PT (black circle) D-PT. (**E**–**H**) Preservation of the hepatic parenchyma (Z3, Z2, Z1, and PT) in NC cases (black circles) E-Z3, F-Z2, G-Z1, H-PT. Z3: centrolobular zone; Z2: midzonal zone; Z1: periportal zone; PT: portal tract. NC: normal control. 400× magnification (scale: 20 µm).

The changes in the histopathological patterns were highly intense in the liver lobules, characterised mainly by macro- and micro-vesicular steatosis, cell swelling (cells are typically swollen, with compression or displacement of adjacent structures), the appearance of apoptotic bodies, and focal points of lytic necrosis (showing absence of intact cell morphology due to membrane lysis), coagulative necrosis (anucleated cells with preserved cell outlines), hyperplasia of Kupffer cells (increase in the number of Kupffer cells), and extensive haemorrhagic foci (Figure 2A–C). Infiltrates of lymphocytes, neutrophils, and macrophages were observed during the inflammatory response in the PT (Figure 2D).

### 3.2. Immunoexpression of Apoptotic Markers

**Figure 3:**
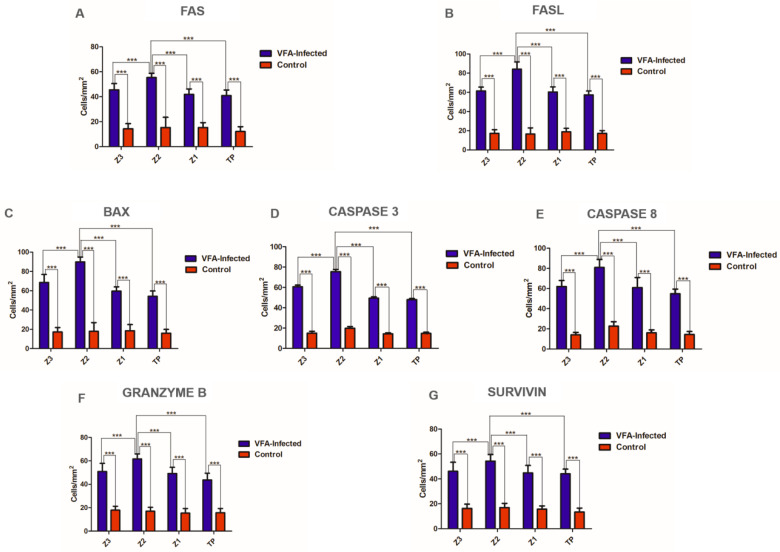
Quantitative analysis of immunohistochemical data positive for (**A**) FAS, (**B**) FASL, (**C**) BAX, (**D**) CASPASE 3, (**E**) CASPASE 8, (**F**) GRANZYME B, and **(G)** SURVIVIN in zones Z3, Z2, Z1, and PT in the hepatic parenchyma of fatal yellow fever cases (blue) and negative controls (red). Z3: centrolobular zone; Z2: midzonal zone; Z1: periportal zone; PT: portal tract; one-way ANOVA; *** *p* < 0.0001. Tukey’s post-hoc test; *** *p* < 0.0001.

**Figure 4:**
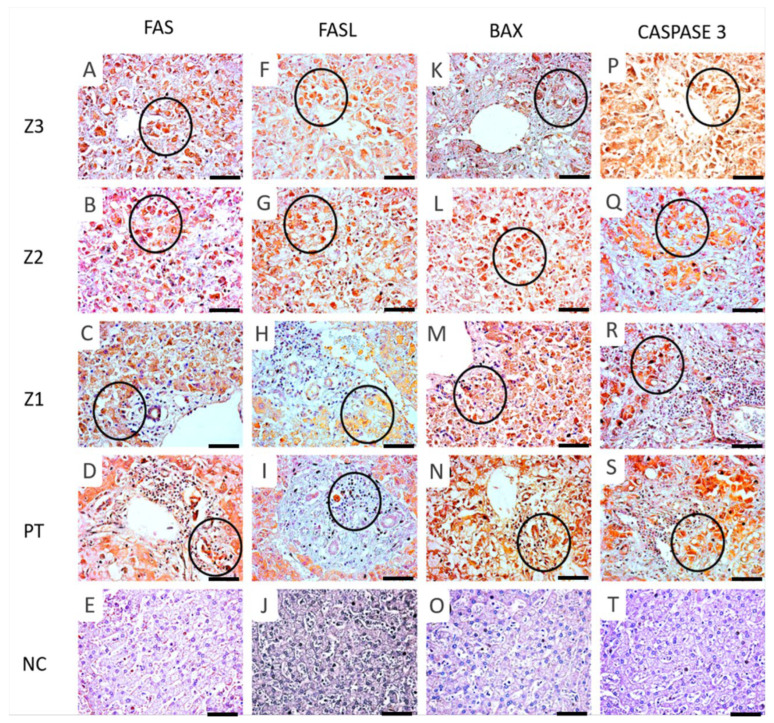
Immunostaining for FAS (**A**–**D**) (Z3-A, Z2-B, Z1-C), FASL (**F**–**I**) (Z3-F, Z2-G, Z1-H), BAX (**K**–**N**) (Z3-K, Z2-L, Z1-M), CASPASE 3 (**P**–**S**) (Z3-P, Z2-Q, Z1-R), in the hepatocytes (black circle) and D-PT, I-PT, N-PT, and S-PT in the inflammatory infiltrates (black circles). Preservation of parenchyma hepatic in control cases and minimal expression of FAS (**E**), FASL (**J**), BAX (**O**), and CASPASE 3 (**T**). 400× magnification (scale: 20 µm). Z3: centrolobular zone; Z2: midzonal zone; Z1: periportal zone; PT: portal tract; NC: normal control.

**Figure 5:**
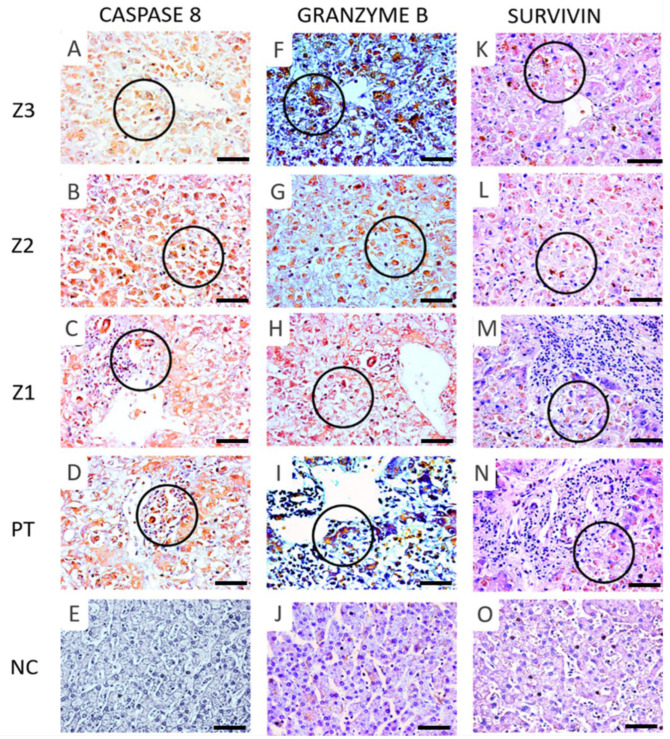
Immunostaining for CASPASE 8 (**A**–**D**) (Z3-A, Z2-B, Z1-C), GRANZYME B (**F**–**I**) (Z3-F, Z2-G, Z1-H), (**K**–**N**) SURVIVIN (Z3-K, Z2-L, Z1-M), in the hepatocytes (black circles) and D-PT, I-PT, N-PT in the inflammatory infiltrates (black circles). Preservation of hepatic parenchyma in control cases and minimal expression of CASPASE 8 (**E**), GRANZYME B (**J**), SURVIVIN (**O**). 400× magnification (scale: 20 µm). Z3: centrolobular zone; Z2: midzonal zone; Z1: periportal zone; PT: portal tract; NC: normal control.

Immunohistochemical analysis of the lobular regions (Z3, Z2, Z1, and PT) tested positive for FAS, FASL, CASPASE 3, CASPASE 8, BAX, FAS, FASL, GRANZYME B, and SURVIVIN, whose expression levels were significantly different compared to those of the controls (Table 2 and Figure 3). All markers showed significantly intense immunostaining in Z2 compared to other lobular regions (*p* < 0.05, Table 2 and Figure 3). Generally, pro and anti-apoptotic markers were predominant in the hepatocytes and inflammatory infiltrates compared to those in the controls (Figure 4 and Figure 5).

## 4. Discussion

In this study, the histopathological investigation suggested that the main alterations in the hepatic lobes were consistent with extensive tissue destruction in the hepatic lobules, characterised by haemorrhagic foci (Figure 2A–C), areas of lytic and coagulative necrosis, and apoptotic bodies. The viral tropism by resident cells and the intense liver damage resulting from a disproportionate immune response directly correlated with the degree of liver parenchymal involvement [13,14,15]. These findings corroborate with those of the previous studies on YF pathogenesis. In this follow-up study, liver cell damage seemingly occurred from a series of mechanisms that cause tissue hypoxia, influencing the stress response of the endoplasmic reticulum and mitochondrial dysfunction [16,17,18]. In the pathophysiology of YFV infection, virus-induced apoptosis is considered one of the immunological escape strategies, modulating the inflammatory process in situ and consequently the organ tissue damage [19]. Understanding protein interactions and their biological significance during viral infection are paramount to correlating the viral pathogenesis mechanism, as viruses use the host cell machinery for their replication [20,21]. Therefore, we focused on the markers involved in the apoptosis of YFV-infected hepatocytes to further elucidate the YF pathogenesis mechanism.

In the liver, severe apoptosis results in acute organ failure, whereas persistent apoptosis is often associated with fibrinogenesis and chronic liver dysfunction [22,23,24]. In addition to YFV, apoptotic cell death has been described in other flaviviral infections such as dengue virus (DENV), Japanese encephalitis virus (JEV), West Nile virus (WNV), and Zika virus (ZIKV) [25,26,27,28]. However, these apoptotic events are not exclusive to flaviviruses. We observed the expression of FAS and FASL proteins in all liver lobes, particularly in hepatocytes, which were higher in the zonal mid-region. The FAS-FASL binding drives a Fas-associated death domain (FADD) protein cluster that recruits CASPASE 8 and CASPASE 10 to form a death-inducing signalling complex (DISC), leading to a mitochondrial-independent cell death signalling cascade. As death receptor-mediated apoptotic signals are usually insufficient to initiate the cascade in hepatocytes, the participation of the mitochondria-mediated pathway is also required for its initiation. This occurs when DISC production is insufficient, causing the activated CASPASE 8 to proteolytically cleave the proapoptotic Bcl-2 homology 3 (BH3) protein of the Bcl-2 family, transforming BH3-interacting domain death agonist (Bid) to tBid, and activating Bcl-2-associated X (Bax) and Bcl-2 homologous antagonist killer (Bak) proteins [29,30,31]. Therefore, this appears to be a mitochondrial apoptotic pathway activated via an extrinsic mechanism in situ, resulting from the death receptor activation in fatal cases of YFV in humans.

Our investigation revealed a high degree of CASPASE 3 and CASPASE 8 expressions, mainly in the Z2 region. In addition, BAX protein immunoexpression was the highest among all our analysed markers, with the highest degree observed in Z2. This may indicate the preference for mitochondrial apoptotic pathways in YFV-infected hepatocytes initially and preferentially mediated by the intrinsic pathway. Notably, in this process, the intrahepatic infiltrating NK and CD8 T cells cause hepatic cell death in different phases of DENV infection. In fatal cases of ZIKV-induced microcephaly, FAS, FASL, and tumour necrosis factor (TNF)-α were also overexpressed and directly regulated the mechanisms of neural parenchymal cell injury [32,33]. The action of pro-inflammatory cytokines tends to generate tissue hypoxia, which in turn increases oxidative stress. In addition, the intrinsic changes in the mitochondrial membrane caused by the Bcl-2 protein family activation may result in the activation of CASPASE 3, which was found to be highly expressed in our study.

We investigated the immunoexpression of GRANZYME B and observed that this protein was expressed in all regions of the lobes, predominantly in the Z2 region, similar to other markers. These results indicate that liver infection by VFA causes liver damage by infiltration of cytotoxic T lymphocytes, which, in addition to expressing FASL on their surface, release GRANZYME B and consequently lead to apoptosis of hepatocytes. GRANZYME B-induced cell death depends on caspases and causes permeabilisation of the mitochondrial outer membrane, mediated by BAX, Bak, and Bid, releasing cytochrome c and other apoptogenic factors into the cytosol. Such mechanisms can also be observed in infections by flaviviruses [10,20,34]. Finally, given that the SURVIVIN protein possesses important anti-apoptotic activity, we also quantified this marker in our samples, which was expressed in all areas of the liver lobes, with the highest expression level being in the Z2 region, compared to the controls. The anti-apoptotic activity of SURVIVIN occurs through the inhibition of effector caspases 3, 7, and 9 [35,36]. Furthermore, this marker is known to inhibit important proteins involved in the activation of caspases; thus, increasing cell survival both directly and indirectly. However, SURVIVIN expression in the environment cannot negatively regulate the progression of cell injury and directly affects the immunopathogenecity of the disease. Collectively, our study demonstrated the complexity of apoptosis in situ caused by YFV infection, and the data suggest that apoptosis in liver parenchymal lesions may significantly contribute to tissue damage in fatal cases of YF in humans. Therefore, additional in vitro and in vivo studies are required to establish the relationship between apoptosis and YFV pathology in the liver.

## Figures and Tables

**Table 1:** Antibodies used to monitor apoptosis in fatal human YF cases.

Markers	Reference	Dilution
BAX	Abcam/ab32503	1:100
SURVIVIN	Novus/NB500-201	1:100
FAS	Dako/m3555	1:100
FASL	Abcam/ab15285	1:100
GRANZYME B	Dako/m7235	1:100
CASPASE 3	Abcam/ab4051	1:100
CASPASE 8	Abcam/ab25901	1:100

**Table 2:** Quantitative analysis of apoptotic markers in the hepatic parenchyma (Z3, Z2, Z1, and PT) in fatal YFV cases.

Markers (Cells/mm^2^)	Z3	Tukey (*p* ≤ 0.05)	Z2	Tukey (*p* ≤ 0.05)	Z1	Tukey (*p* ≤ 0.05)	TP	Tukey (*p* ≤ 0.05)	ANOVA (*p* ≤ 0.05)
**BAX**	68.53 ± 8.36	***	89.9 ± 4.9	***	59.64 ± 4.37	***	54.24 ± 5.54	***	***
**Control**	17.28 ± 4.5		17.9 ± 9.0		18.56 ± 6.36		16.0 ± 3.9		
**GRANZYME B**	50.92 ± 6.9	***	61.64 ± 4.25	***	49.22 ± 5.35	***	43.7 ± 5.7	***	***
**Control**	17.92 ± 3.2		16.96 ± 3.3		15.36 ± 3.85		15.68 ± 3.64		
**FAS**	45.56 ± 4.99	***	55.38 ± 3.3	***	41.9 ± 4.3	***	40.97 ± 4.4	***	***
**Control**	14.4 ± 4.0		15.36 ± 8.2		15.36 ± 3.8		12.16 ± 3.8		
**FASL**	61.56 ± 3.9	***	84.13 ± 7.5	***	60.34 ± 5.3	***	57.37 ± 4.0	***	***
**Control**	17.28 ± 3.8		16.64 ± 6.3		18.8 ± 3.6		17.2 ± 2.8		
**SURVIVIN**	46.1 ± 7.2	***	54.32 ± 5.2	***	44.8 ± 6.0	***	44.11 ± 3.7	***	***
**Control**	16.28 ± 3.4		16.96 ± 3.3		15.68 ± 2.6		13.44 ± 3.1		
**CASPASE 3**	60.65 ± 7.6	***	75.57 ± 9.9	***	49.45 ± 6.1	***	48.06 ± 4.7	***	***
**Control**	15.0 ± 4.0		19.8 ± 3.8		14.4 ± 2.2		14.76 ± 2.8		
**CASPASE 8**	61.94 ± 6.0	***	80.91 ± 7.9	***	60.95 ± 9.9	***	54.78 ± 4.6	***	***
**Control**	14.08 ± 2.3		22.72 ± 4.2		16.0 ± 2.9		14.4 ± 2.9		

One-way ANOVA; *** *p* < 0.0001. Tukey’s post-hoc test; *** *p* < 0001.

## Data Availability

The data used to support the fidings of this study are included within the article.
